# Downregulation of *yidC* in *Escherichia coli* by Antisense RNA Expression Results in Sensitization to Antibacterial Essential Oils Eugenol and Carvacrol

**DOI:** 10.1371/journal.pone.0057370

**Published:** 2013-03-04

**Authors:** Supriya Deepak Patil, Rajnikant Sharma, Santosh Srivastava, Naveen Kumar Navani, Ranjana Pathania

**Affiliations:** Chemical Biology and Drug Discovery Lab, Department of Biotechnology, Indian Institute of Technology Roorkee, Roorkee, Uttarakhand, India; University of Cambridge, United Kingdom

## Abstract

**Background:**

The rising drug resistance in pathogenic bacteria and inefficiency of current antibiotics to meet clinical requirements has augmented the need to establish new and innovative approaches for antibacterial drug discovery involving identification of novel antibacterial targets and inhibitors. Being obligatory for bacterial growth, essential gene products are considered vital as drug targets. The bacterial protein YidC is highly conserved among pathogens and is essential for membrane protein insertion due to which it holds immense potential as a promising target for antibacterial therapy.

**Methods/Principal Findings:**

The aim of this study was to explore the feasibility and efficacy of expressed antisense-mediated gene silencing for specific downregulation of *yidC* in *Escherichia coli*. We induced RNA silencing of *yidC* which resulted in impaired growth of the host cells. This was followed by a search for antibacterial compounds sensitizing the YidC depleted cells as they may act as inhibitors of the essential protein or its products. The present findings affirm that reduction of YidC synthesis results in bacterial growth retardation, which warrants the use of this enzyme as a viable target in search of novel antibacterial agents. Moreover, *yidC* antisense expression in *E. coli* resulted in sensitization to antibacterial essential oils eugenol and carvacrol. Fractional Inhibitory Concentration Indices (FICIs) point towards high level of synergy between *yidC* silencing and eugenol/carvacrol treatment. Finally, as there are no known *YidC* inhibitors, the RNA silencing approach applied in this study put forward rapid means to screen novel potential *YidC* inhibitors.

**Conclusions/Significance:**

The present results suggest that YidC is a promising candidate target for screening antibacterial agents. High level of synergy reported here between *yidC* silencing and eugenol/carvacrol treatment is indicative of a potential antibacterial therapy. This is the first report indicating that the essential gene *yidC* is a therapeutic target of the antibacterial essential oils eugenol and carvacrol in *E. coli*.

## Introduction

Evolution of multidrug resistance in bacterial pathogens has led to a situation in which there are diminutive or no treatment options for infections with certain pathogens. Consequently, the need for new antibacterial agents has increased [1]–[4]. Regardless of the rise of resistant pathogens, the pace of new antibiotic approvals is falling [1], [5], [6]. Over the past few decades, in order to solve the problem of drug resistance, existing antibiotics are being chemically modified [1]. However, this has led to relatively narrow diversity of compound classes aiming at limited number of targets [7]. Therefore, the solution lies in isolating or synthesizing new antibacterial agents with non-classical cellular targets. Antimicrobials that target unexplored proteins in bacteria are not likely to be vulnerable to existing target-based mechanisms of resistance because of their distinctive modes of action [8].

Most of the antibiotics that are in current medical use were identified in whole-cell (phenotype based) screening assays in which compounds that show potent antibacterial activity are selected first, followed by identification of their cellular targets [2], [6]. However, this has consequently led to rediscovery and identification of same classes of antibiotics which proved inadequate to beat the problem of drug resistance. Moreover, it is a difficult task to deduce targets and mode of action of antibacterial agents identified in random whole cell screens. Therefore, target-directed antibacterial discovery approaches should be created by merging random whole-cell screening with genetic methods. Differential growth assays based on bacterial antisense technology can be applied for target-directed whole-cell screening [2], [9]. Antisense expression approach can be used to down-regulate expression of a specific gene at the post-transcriptional level by means of an inducible promoter and a plasmid vector containing the gene fragment in antisense orientation. If the gene is essential, antisense RNA (asRNA) synthesis will lead to inhibition of host cell growth by binding to the corresponding messenger RNA (mRNA) and making it non-functional [10], [11]. This is achieved largely by increasing degradation of mRNA [11] as well as inhibition of translation by hybridizing to sequences flanking the ribosome binding site (RBS) and the start codon of the mRNA [12], [13]. Moreover, a surplus relative availability of asRNAs will probably facilitate them to out-compete target mRNAs for ribosome [10]. Using this approach in *Staphylococcus aureus*, several inhibitors targeting essential enzymes of fatty acid synthesis pathway were discovered [9], [14], [15]. Although asRNA approach has been used to elaborate the mechanism of action of natural products in *E. coli* [16], there is a dearth of published reports describing the application of this approach for screening target specific antibacterial agents in Gram-negative bacteria.

Proteins that are mandatory for bacterial survival and are conserved among pathogens are considered excellent targets for inhibitor screening [2], [6]. The availability of complete genome sequences allows us to identify proteins that are conserved across the medically important pathogens but absent in higher eukaryotes thereby excluding potential toxicity [2]. Many essential proteins have been identified in bacteria as prospective drug targets [17], [18]. However, the current drugs target only a few bacterial pathways [19]. Conversely, identification of new classes of antibacterial compounds which are directed towards unexplored targets in bacteria is critical to combat emerging resistance [8]. One of the essential *E. coli* proteins, YidC is a 60-kDa membrane protein [20]. Its functions involve membrane protein translocation and insertion. YidC functions jointly with the Sec translocase but can also function separately to facilitate the insertion of proteins into the cell membrane [21]. It is an evolutionarily conserved protein and has been shown experimentally to be essential for growth in *E. coli* [22]. It is shown that YidC depletion leads to decrease in the functional assembly of cytochrome *o* oxidase and F_1_F_o_ ATPase along with a strong reduction of proton-motive force in *E. coli* [23]. All Gram-positive bacteria have at least one gene encoding a YidC homolog within their genome, but most, like the oral pathogenic bacterium *Streptococcus mutans* also contain a second gene for a YidC homolog [24]. Being a common feature of both Gram-negative and Gram-positive bacteria, it is an attractive target for the development of broad-spectrum antibacterial agents. Additionally, YidC is different from targets of existing drugs [19], is essential for bacterial growth [22] and exhibits divergence in eukaryotes and bacteria [25] which further sustain its intrinsic significance as a promising antibacterial target.

Natural products have been an affluent resource of leads for antibacterial drug discovery [3]. Drugs originated from plant sources have been used for healing various human ailments for thousands of years in the form of traditional medicines [7]. Essential oils, also known as volatile oils are extracted from plants. They are aromatic and volatile in nature and many of them are known to possess antibacterial properties [26]. Eugenol and carvacrol are antibacterial components of several essential oils which are known to inhibit a number of Gram-negative and Gram-positive pathogenic bacteria [27]. They kill bacteria by causing membrane disruption and hence increased permeability of bacterial cells. They are also known to possess membrane bound ATPase inhibitory activity in *E. coli* [28].

The aim of this study was to explore the feasibility and efficacy of expressed antisense-mediated gene silencing for specific downregulation of *yidC* in *E. coli*. An antisense expression vector, pHN678 was used, that is designed such that expressed asRNA molecules have paired termini to increase their stability and hence gene silencing effectiveness in *E. coli* [10]. By using this approach, it was possible to decrease the cellular level of *yidC* mRNA consequently leading to retarded growth of host cells. Reduction of expression of a gene can sensitize cells to compounds that inhibit the activity of that gene product or related process [16], [29]. Our results show that *yidC* antisense expressing clone is sensitized to membrane disintegrating and membrane bound ATPase inhibitory antibacterial essential oils eugenol and carvacrol. To the best of our knowledge, this is the first report which indicates that the essential gene *yidC* is a therapeutic target of antibacterial essential oils eugenol and carvacrol.

## Materials and Methods

### Strains and Plasmids

Antisense cloning vector pHN678 was kindly provided by Dr Liam Good (Department of Pathology and Infectious Diseases, Royal Veterinary College, London, UK). The multiple cloning site (MCS) of pHN678 is flanked by paired-termini to impart stability to antisense RNA after expression [10]. *E. coli* DH5α strain was used as a host cell throughout the experiments and grown in Luria Bertani (LB) medium (Merck, Germany) at 37°C and 250 rpm shaking if not otherwise specified. Chloramphenicol (Sigma Chemical Company, St. Louis, Mo, USA) at a concentration of 30 µg/ml was included to maintain the plasmid.

### Transcript Target Site Selection

Part of the *yidC* gene sequence for antisense expression was chosen based on criteria described previously [30]. It has been shown earlier that an antisense sequence should have small length, should be located around the start codon to include promoter and coding regions and should have minimal secondary structures. The secondary structures were predicted by RNAfold (http://rna.tbi.univie.ac.at/cgi-bin/RNAfold.cgi). The primers were designed such that the expressed asRNAs hybridize to the sequences flanking the RBS and the start codon of *yidC* mRNA. The primers used in this study are given in table 1.

**Table 1:** Primers used in this study.

Primer	Primer sequence (5′–3′)	Target	Product size(base pair)	Purpose of amplicon	Reference/Source
yidC-XF	cgtccatggcatgaaagacacgaacag	Antisense of *yidC*	134	Cloning of *yidC* antisense into pHN678	This study
yidC-XR	tgtctcgaggtattaaaatgccacc				
murA-XF	cgtccatgggagcagcatttttagcgc	Antisense of *murA*	130	Cloning of *murA* antisense into pHN678	[30]
murA-XR	tgtctcgaggctatgggcgattcgc				
yidC-F	ctggggcttctccattatca	*yidC* gene fragment	279	qPCR of *yidC*	This study
yidC-R	agttcaacggaacccatcag				
16SrRNA-F	cagccacactggaactgaga	16S rRNA gene fragment	204	qPCR of reference gene	This study
16SrRNA-R	gttagccggtgcttcttctg				

### Cloning of Antisense RNA Expression Constructs, Insert DNA Sequencing and Bioinformatic Analysis

Selected nucleotide sequence of *yidC* and *murA* essential genes were amplified from *E. coli* MG1655 genomic DNA (using primers given in table 1), digested with *Nco*I (Fermentas, USA) and *Xho*I (Fermentas, USA) and cloned into the MCS of pHN678 with similarly digested ends. Recombinant plasmids were transformed in *E. coli* DH5α competent cells and selected on LB agar supplemented with chloramphenicol (30 µg/ml).

In order to confirm inserts, plasmids were isolated from the transformants and sequenced at Ocimum Biosolutions, India to determine the DNA sequences of the inserts and their orientations. The DNA sequences were then compared to the annotated genomic sequence of *E. coli* MG1655 (Genbank accession number NC_000913) to determine the origin of DNA inserts and their orientation using BLAST (Basic Local Alignment Search Tool) from NCBI (National Center for Biotechnology Information) (http://blast.ncbi.nlm.nih.gov/Blast.cgi).

### *E. coli* Growth Inhibition Experiments

The growth inhibitory effect of antisense expression of *yidC* and *murA* essential genes (antisense expression of *murA* was used as a positive control) on *E.coli* was examined by assessing cell growth. *E. coli* DH5α carrying pHN678, pHN678 with an *yidC* antisense insert (pHN678-yidC) or pHN678 with an *murA* antisense insert (pHN678-murA) were grown overnight in 10 ml of LB broth supplemented with chloramphenicol (30 µg/ml) at 37°C with shaking at 250 rpm. Following overnight growth, the cells were diluted 100 fold in fresh LB broth medium supplemented with chloramphenicol (30 µg/ml). This diluted culture (5 µl) was spotted on LB chloramphenicol plates (for uninduced conditions) and LB chloramphenicol plates supplemented with Isopropyl-beta-thiogalactopyranoside (IPTG) (Fermentas, USA) concentrations ranging from 1 to 20 mM (for induced conditions). These plates were incubated at 37°C for 14 hours. Growth on uninduced and induced plates was compared with naked eyes.

Antisense expression was also checked in liquid medium as described previously [30] with some modifications. Various concentrations of IPTG (40, 80, 160 and 320 µM) were added in triplicates to Mueller Hinton (MH) broth (Merck, Germany) supplemented with chloramphenicol (30 µg/ml) in separate wells of 96-well plate (Genaxy, India) making up final volume to 100 µl. A control without any IPTG was also included. Overnight cultures of *E. coli* DH5α carrying plasmids expressing asRNA (Table 2) in MH broth supplemented with chloramphenicol (30 µg/ml) were subcultured (1% inoculum) in fresh medium and incubated further till optical density at 600 nm (OD_600_) reached 0.5 to 0.7. This culture was then diluted and standardized by OD_600_ readings to approximately 10^5^ cfu/ml and then 100 µl of this diluted culture was added to each well of a 96-well plate to make a final volume of 200 µl and incubated at 37°C with 150 rpm shaking. The growth was monitored by OD_600_ readings every hour using spectramax plus plate reader (molecular devices, USA).

**Table 2:** Plasmids used in the study.

Plasmid	Description and resistance	Purpose	Antisense target location*and length	Reference/Source
pHN678	IPTG inducible promoter (Ptrc),paired-termini flanking MCS, Cam^R^	Antisense RNA expression vector	n/a	[10]
pHN678-murA	pHN678 derivative, IPTG-inducible promoter (Ptrc), Cam^R^, *murA* antisense insert	Inducible expression of *murA* antisense	−54 to +76 of *murA* (130 nt.)	[30]
pHN678-yidC	pHN678 derivative, IPTG-inducible promoter (Ptrc), Cam^R^, *yidC* antisense insert	Inducible expression of *yidC* antisense	−80 to +54 of *yidC* (134 nt)	This work

* antisense target locations are specified relative to the start codon.

### Relative Real-time (RT) Quantitative PCR (qPCR)

Total RNA was isolated from the cells using TRI reagent (MRC, Cincinnati, Ohio, USA) as per manufacturer’s instructions which was followed by DNase I (Fermentas, USA) treatment for removal of contaminating genomic DNA. Total RNA (1 µg) was converted to strand-specific cDNA in a 25 µl reaction consisting of 1 X RT reaction buffer, 4 mM of each dNTP, 1.6 µM gene specific reverse primers, 20U RNase inhibitor, and 1 µl of lmProm-II Reverse Transcriptase (Promega, Madison, USA). Each 25 µl of PCR reaction contained 12.5 µl of Light cycler 480 SYBR Green I master (Roche diagnostics, Germany), 0.2 µM of each primer and 2 µl (450 ng) of cDNA. Relative qPCR was carried out on Smart Cycler (Cepheid, Sunnyvale, CA) with primers yidC-F/R specific for *yidC* as the target gene, and primers 16S rRNA-F/R specific for 16S rRNA as the reference gene (endogenous gene) (Table 1). From the fluorescent values obtained through the real-time PCR analysis, the threshold cycles (C_T_-value) were obtained. The relative expression ratio was calculated as given below and described previously [31].

Here, ΔC_T_ target gene (control−test) = C_T_ value for the *yidC* gene with the control RNA (RNA from uninduced cells)−C_T_ value of the *yidC* gene with the test RNA (RNA from IPTG induced cells). Also, ΔC_T_ endogenous gene (control−test) = C_T_ value of the 16S rRNA gene with the control RNA (RNA from uninduced cells)−C_T_ value of the 16S rRNA gene from the test RNA (RNA from IPTG induced cells). All samples were analyzed in triplicate. Quantitation of target *yidC* mRNA was normalized against 16S rRNA mRNA and calculated relative to the untreated sample. Mean values with error bars, representing standard deviation from three experimental replicates, were plotted against IPTG doses.

### Minimum Inhibitory Concentration (MIC) Determination

All the antibiotics as well as plant natural products including berberine, piperine, α-terpineol, γ-terpinene, eugenol, carvacrol, aloe emodin, rutin, apigenin and gallic acid were purchased from Sigma-Aldrich, USA. Each was added in a series of dilutions in MH broth to 96-well plates in triplicates to make a final volume of 100 µl. Then, 100 µl of MH broth containing approximately 10^5^ cfu/ml of *E. coli* cells prepared as in growth inhibition experiments was added to these dilutions. MIC was deduced as the lowest concentration of an antimicrobial agent at which the growth of the tested *E. coli* strain was inhibited completely after 12 hours of incubation at 37°C.

### Determining Sensitization and Fractional Inhibitory Concentration Indices (FICIs) for Synergy Studies

Cell-based assays in MH broth as described for growth inhibition experiments were performed against a panel of known antibiotics of different chemical classes and plant natural products with antibacterial activity to obtain fold sensitization values [the ratio between the IC_50_ value (the concentration at which cell growth inhibited 50% compared to control) under non-induced condition and that of induced condition] [32]. Various antibiotics belonging to six different classes based on their mode of action namely cell wall synthesis inhibition (vancomycin and phosphomycin), protein synthesis inhibition (kanamycin and hygromycin), nucleic acid synthesis inhibition (mitomycin and novobiocin), cell membrane disruption (polymyxin B and colistin), metabolic antagonism (trimethoprim) and fatty acid synthesis inhibition (triclosan) as well as some plant natural products (berberine, piperine, α-terpineol, γ-terpinene, eugenol, carvacrol, aloe emodin, gallic acid, rutin and apigenin) were screened for sensitization of YidC depleted strain of *E. coli* as compared to control cells not expressing *yidC* antisense. The FICI values for synergy were calculated as described previously [16]. Synergy is defined by FICI values ≤0.5 [33].

## Results

### Transcript Target Site Selection and Cloning of Antisense RNA Expression Constructs

The fragment of *yidC* sequence chosen for antisense expression consisted of nucleotides in the region from −80 to +54 (134 nt (nucleotides)) as this sequence was found to produce minimum secondary structures as compared to other short sequences flanking the *yidC* start codon. The *yidC* and *murA* antisense constructs were confirmed by sequencing of the plasmids isolated from transformants and BLAST analysis of obtained sequences.

### The Growth Inhibitory Effect of yidC Antisense Expression on Bacterial Cells

**Figure 1:**
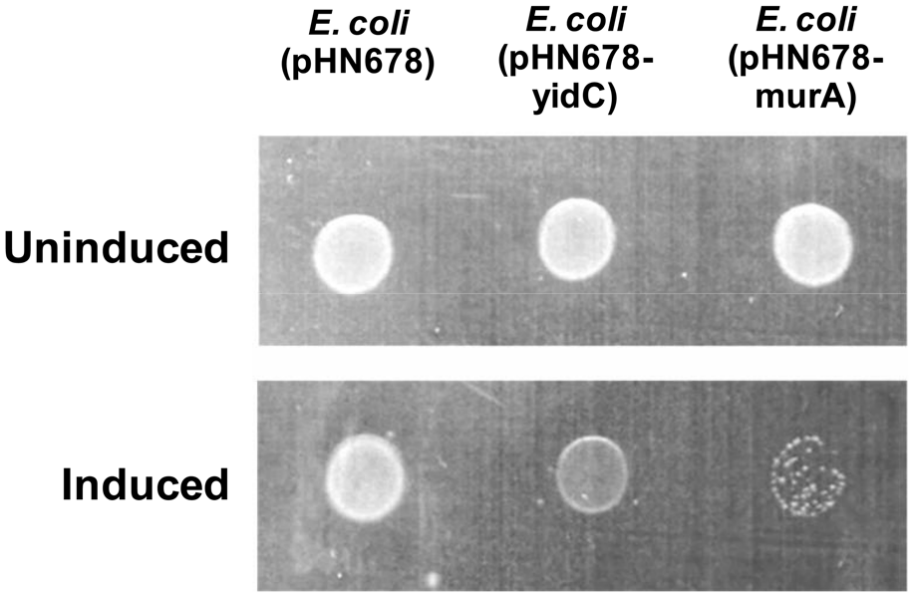
Effect of *yidC* antisense expression on growth of *E. coli*. *E. coli* DH5α harbouring pHN678 (negative control for antisense expression), pHN678-yidC or pHN678-murA (positive control for antisense expression) were cultured in LB broth supplemented with chloramphenicol (30 µg/ml). The overnight cultures were diluted hundred fold in fresh LB broth supplemented with chloramphenicol (30 µg/ml) of which 5 µl was spotted on LB chloramphenicol plates without (upper panel) and with IPTG (lower panel). There was no change observed in the phenotype of *E. coli* DH5α harbouring vector (pHN678) without any insert upon induction with IPTG after 14 hrs of incubation at 37°C. However, the growth of *E. coli* DH5α cells expressing antisense of *murA* and *yidC* showed growth suppression upon induction.

**Figure 2:**
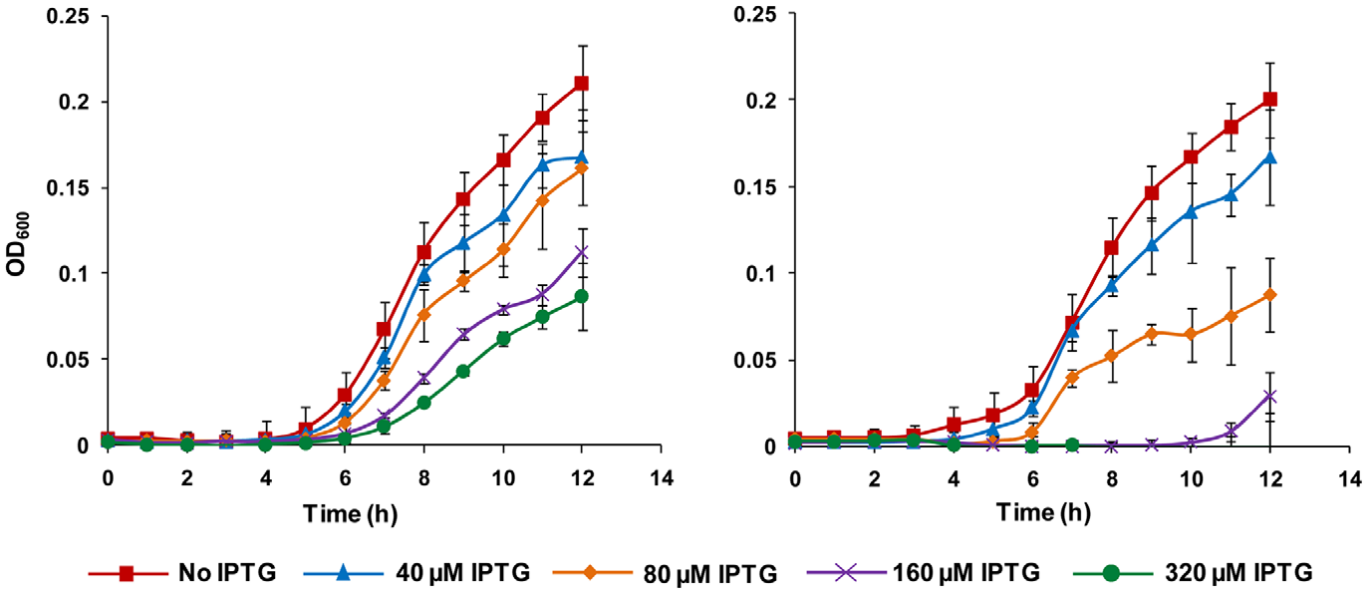
Effect of IPTG concentration on growth of *E. coli* DH5α harbouring *yidC* antisense expressing vector (pHN678-yidC). Graph on left represents growth of *E. coli* DH5α harbouring *yidC* antisense expressing vector (pHN678-yidC) while graph on right represents growth of *E. coli* DH5α harbouring *murA* antisense expressing vector (pHN678-murA) used as positive control. Antisense of the essential genes was induced by IPTG at different concentrations (40, 80, 160 and 320 µM). Addition of IPTG had a clear inhibitory effect on the growth of *E. coli* DH5α cells harbouring antisense inserts which increased with increasing IPTG concentration.

The growth inhibitory effect of *yidC* antisense expression on bacterial cells was examined by assessing cell growth (Figure 1 and 2). Figure 1 shows that addition of IPTG has a clear inhibitory effect on the growth of *yidC* and *murA* antisense expressing *E. coli* DH5α cells due to silencing of genes essential for growth. However, *E. coli* DH5α harbouring pHN678 without any antisense insert showed similar growth on uninduced as well as induced plate. The growth curves for different levels of *yidC* and *murA* asRNA expression are presented in figure 2 which shows that silencing of *yidC* and *murA* lowered the final OD_600_ of *E. coli* cells.

### Effect of *yidC* Antisense Expression on Corresponding Gene Transcripts

**Figure 3:**
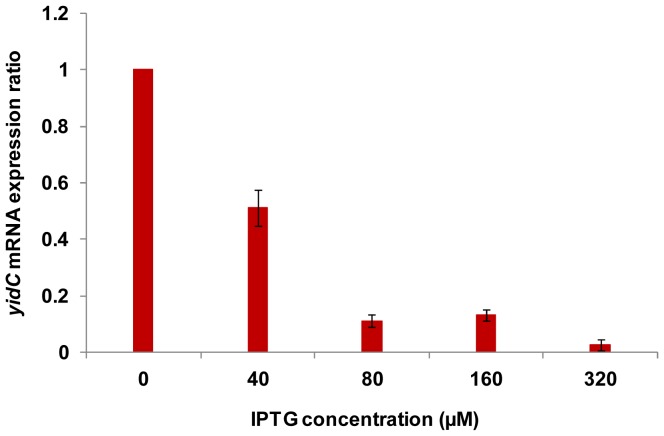
Effect of *yidC* antisense expression on corresponding gene transcript. Relative Real-time quantitative PCR (qPCR) was carried out to compare *yidC* mRNA expression in uninduced and induced pHN678-yidC harbouring *E. coli* DH5α cells. The CT values were averaged from triplicate samples and the expression ratio was calculated. Comparison of expression ratio of *yidC* gene upon its antisense induction in *E. coli* with different IPTG concentrations showed that *yidC* mRNA expression decreased with increasing IPTG concentration. However, the expression is almost same in cells treated with 80 and 160 µM IPTG.

The reduction in growth of bacterial cells upon *yidC* antisense expression could be caused by a decay of specific transcripts of gene *yidC* through antisense mechanism and subsequent reduced expression of protein YidC. We used relative qPCR to compare mRNA expression in uninduced and induced *E. coli* cells harbouring pHN678-yidC and assumed it as a proxy to protein measure in bacteria [34]. We observed that *yidC* mRNA expression diminished progressively with increase in IPTG concentration (figure 3) used for induction. However, the expression is almost same in cells treated with 80 and 160 µM IPTG. The level of the unrelated, constitutively expressed 16S rRNA gene transcript, assayed as a control RNA, did not change under any of the culture conditions as revealed from C_T_ values (data not shown).

### Susceptibility Testing to Various Antibiotics with known Modes of Action

**Figure 4:**
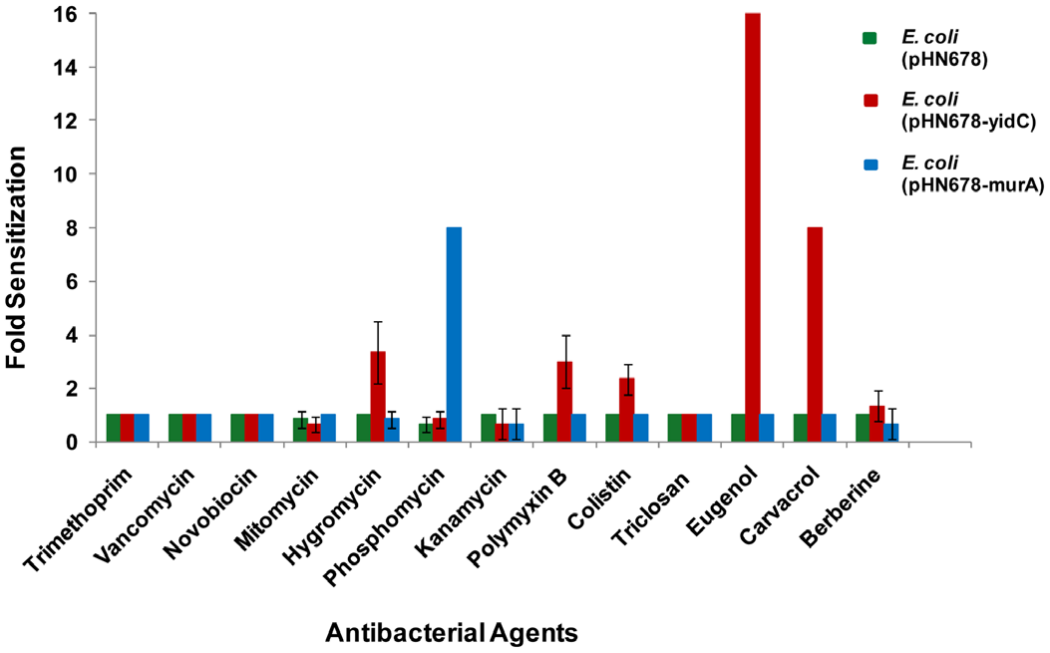
Fold sensitization of *yidC* antisense expressing *E. coli* DH5α cells to various antibacterial agents. Various antibiotics belonging to six different classes based on their mode of action namely cell wall synthesis inhibition (vancomycin and phosphomycin), protein synthesis inhibition (kanamycin and hygromycin), nucleic acid synthesis inhibition (mitomycin and novobiocin), cell membrane disruption (polymyxin B and colistin), metabolic antagonism (trimethoprim) and fatty acid synthesis inhibition (triclosan) as well as some plant natural products such as membrane bound ATPase activity inhibiting essential oils eugenol and carvacrol, cell division protein FtsZ inhibiting alkaloid berberine as well as some other plant natural products named in materials and methods (data not shown) were screened for sensitization of YidC depleted strain of *E. coli* DH5α as compared to control cells (*E. coli* DH5α harbouring only vector or expressing antisense of *murA* essential gene). Fold sensitization represents the change in antibiotic IC50 values upon antisense induction. *E. coli* DH5α cells expressing *yidC* antisense showed considerable sensitization to eugenol (16 fold) and carvacrol (8 fold) as compared to control cells. However other natural products and antibiotics with known mode of action did not result in fold sensitization equal to or greater than that of *murA* antisense expressing E. coli cells to phosphomycin (positive control).

The sensitization of *yidC* asRNA expressing *E. coli* cells towards various antibiotics with known mode of action was studied in cell-based assays. We used *murA* asRNA expressing clone as a positive control for susceptibility testing because phosphomycin is a well established inhibitor of MurA. Induction with IPTG rendered *E. coli* cells expressing *murA* antisense more susceptible to phosphomycin (apparent from MIC which decreased from 62.5 ng/ml in case of control cells harbouring pHN678 alone to 7.8 ng/ml in case of cells harbouring pHN678-murA antisense construct). Our results indicate that at 70 µM IPTG, the *murA* asRNA expressing clone exhibits 8 fold increase (IC_50_ at 0 µM divided by IC_50_ at 70 µM IPTG) in sensitivity to phosphomycin (Figure 4). The optimized cell-based assay was performed against serial dilutions of nine other antibiotics. Results showed that the *yidC* asRNA clone did not show comparable sensitization to any of the antibiotics tested (Figure 4).

### Sensitization of *E. coli* Expressing yidC Antisense to Plant Derived Antibacterial Natural Products

**Figure 5:**
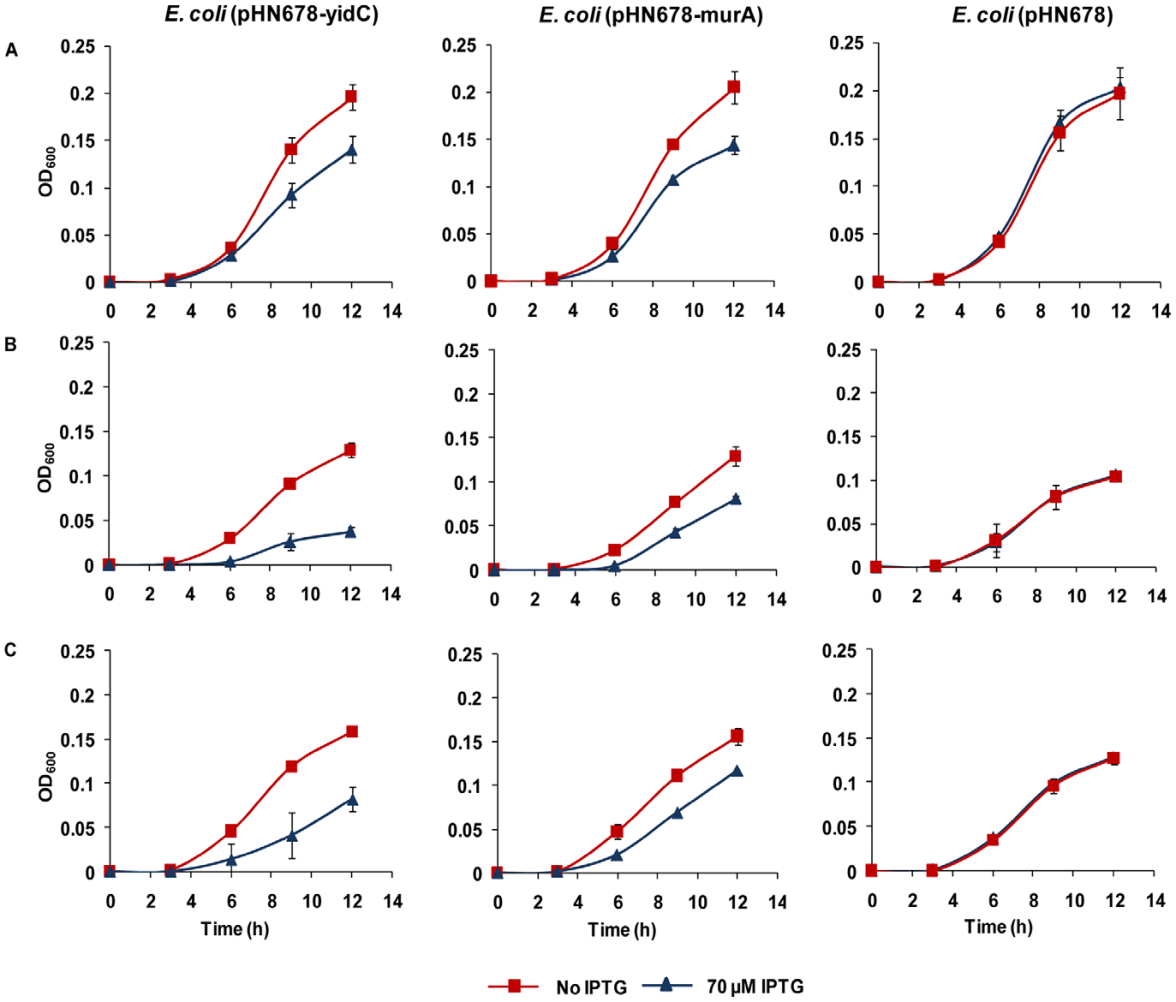
Effect of antisense RNA mediated *yidC* silencing on *E. coli* susceptibility to eugenol and carvacrol. Growth curves for *E. coli* DH5α carrying the control (pHN678 and pHN678-murA) and pHN678-yidC plasmids. (A) In absence of any antibacterial agent. (B) In presence of sub lethal concentration of eugenol. (C) In presence of sub lethal concentration of carvacrol. The growth curve experiment was carried out as described in materials and methods. Antisense expression was induced by 70 µM IPTG. Data are the means ± standard deviations of the experiment performed in triplicate.

*E. coli* growth was monitored both in the presence and absence plant natural products (berberine, piperine, α-terpineol, γ-terpinene, eugenol, carvacrol, aloe emodin, rutin, apigenin and gallic acid) with or without *yidC* silencing by asRNA expression. Silencing of *yidC* caused the bacteria to be more sensitive to eugenol and carvacrol (Figure 4 and 5). Upon *yidC* asRNA expression, bacterial growth was completely inhibited at a lower concentration of the essential oils eugenol and carvacrol as compared to cells not expressing *yidC* asRNA. Declined YidC expression resulted in a reduction of the MIC for eugenol from 2.5 mg/ml without IPTG to 0.156 mg/ml for 70 µM IPTG. Similarly the MIC for carvacrol was reduced from 0.3 mg/ml to 0.03 mg/ml upon induction with 70 µM IPTG. However, the control strains harbouring either vector pHN678 without any insert or pHN678-murA antisense construct were not affected. In addition, the calculated FIC indices revealed synergy between *yidC* asRNA expression and eugenol (FICI = 0.13) and carvacrol treatment (FICI = 0.2) and suggested that no synergy existed between eugenol/carvacrol addition and silencing of the control gene *murA* (FICI = 1).

## Discussion

Rapid evolution of drug resistance mechanisms among bacterial pathogens has unfortunately exceeded our capacity to develop new antibiotics. Novel antibacterial drug screening strategies based on gene silencing by asRNA expression can competently make available drugs to battle emerging infections. A strain expressing asRNA directed towards specific gene transcript will have a reduced pool of cognate protein and hence will be sensitized to inhibitors of that protein or its products as shown in previous studies [16]. Antisense based RNA silencing is highly specific and the specificity has been exemplified by various methods like western blotting [35], complementation of the target gene, qRT-PCR [30] etc. It has been demonstrated that *S. aureus* strain expressing asRNA directed towards *fabF* was sensitized 12-fold to cerulenin, a FabF inhibitor, as compared to uninduced cells not expressing asRNA. Moreover the strain was not sensitive to other antibiotics [36]. Similarly in *E. coli*, it was demonstrated that cells expressing antisense of *fabI* were sensitized to FabI inhibitor triclosan but not trimethoprim (inhibitor of dihydrofolate reductase) or rifampicin (inhibitor of RNA polymerase) [10]. Due to extreme sensitivity of asRNA expressing strain towards specific inhibitors, RNA-silencing based screens enable detection of compounds (such as natural product secondary metabolites which are often produced in small quantities [37]) that would have gone undetected in traditional whole cell based antibacterial screening. The applicability of this approach to discover novel antibacterial agents is proved in *S. aureus* against which several natural product inhibitors of enzymes of fatty acid synthesis pathway like platensimycin [15], platencin [14], phomallenic acids [9] etc. were discovered by using this approach. Thus, the target specific whole cell screening based on asRNA expression is a promising approach for antibacterial drug discovery. Although asRNA based gene silencing approach has been used to elaborate the mechanism of action of berberine in *E. coli* [16], published reports describing the application of this approach for screening target specific antibacterial agents in Gram-negative bacteria are lacking.

YidC, a bacterial membrane integral protein is of immense interest as a potential target for antibacterial therapy due to its conservation in pathogens and essentiality for host survival [22]. The *yidC* gene encoding this *E. coli* membrane insertase is highly conserved among several Gram-negative pathogens. Sequence alignment of *E. coli yidC* by BLAST showed 99% gene similarity in *Shigella flexneri* and *Shigella dysenteriae*, 91% gene similarity in *Citrobacter koseri*, 90% gene similarity in *Salmonella bongori*, *Salmonella enterica* subsp. *enterica* serovar Typhi and *Salmonella enterica* subsp. *enterica* serovar Typhimurium and 89% gene similarity in *Enterobacter cloacae*. No inhibitors of YidC have been reported so far. Therefore, we set out to target *yidC* in *E. coli* by asRNA expression to confirm the effect of its downregulation on growth of *E. coli*. Our results show that the expression of antisense of −80 to +54 nt of *yidC* results in diseased phenotype of host *E. coli* cells. Further, the growth inhibition effect of the *yidC* antisense expression is due to the decay of *yidC* mRNA transcript which evidently results in decrease of YidC synthesis and hence the diseased phenotype of host cells.

The plant-derived essential oils, eugenol and carvacrol have a long history of use as flavouring agents and food preservatives due to their antimicrobial activity. These compounds have been classified as GRAS (generally recognized as safe) by the Food and Drug Administration (FDA), agency of the United States Department of Health and Human Services (Carvacrol: 21CFR172.515; Eugenol: 21CFR582.60). Eugenol is a major component (approximately 85%) of clove oil while carvacrol is one of the major components of oregano and thyme oils [38]. These molecules are known to disintegrate bacterial membrane and inhibit the membrane bound ATPase activity of *E. coli* cells [28]. It has been shown that interactions between mRNA- and protein-level inhibitors directed towards the same genetic target can be synergistic [39]. Therefore, due to the membrane bound ATPase inhibitory activity of eugenol and carvacrol, they were checked for synergy with YidC depletion as F_o_c (membrane subunit of the F1Fo ATPase) is a substrate for YidC owing to which YidC depletion causes adverse effect in assembly of functional membrane bound ATPases [23]. Additionally, for comparison, other plant natural products including alkaloids like berberine and piperine; essential oil components like α-terpineol and γ-terpinene; anthraquinone like aloe emodin; polyphenol like gallic acid and flavanoids like rutin and apigenin were screened for sensitization. Here we report a novel and important observation that downregulation of *yidC* causes *E. coli* to become more susceptible to inhibition by eugenol and carvacrol. In the control assay, upon silencing of another essential gene, *murA*, sensitization to eugenol or carvacrol was not observed while sensitization to phosphomycin was apparent. MurA (UDP-*N*-acetylglucosamine enolpyruvyl transferase) is the well established target of phosphomycin in *E. coli* [40]. On the basis of the derived FICI values and the results from growth curves, it is clear that synergy exists between *yidC* silencing and the essential oils eugenol and carvacrol treatment. We hypothesize that the synergetic effect is due to the reason that YidC depletion results in decline of functional membrane bound ATPases, the activity of which is further inhibited by essential oils eugenol and carvacrol. Our study shows that *yidC* depleted cells do not show comparable sensitization to several antibiotics with different modes of action indicating that drugs targeting YidC are unlikely to be susceptible to resistance mechanisms which bacteria use to defeat current antibiotic classes.

A complementary gene dosage based approach for identifying drug-target interactions is multicopy suppression profiling. In this approach, a specific target protein is over expressed such that it exceeds the quantity of antibacterial molecules leading to suppression of growth inhibitory phenotype. This approach has been successfully applied in *E. coli* to determine mode of action of an antibacterial small molecule along with identification of established targets of various well known antibiotics [41]. The fact that multicopy suppression measurement with target protein overexpression can be used to determine target of an antibacterial molecule indicate that a similar concept can be used to design whole cell assays to screen target specific inhibitors. However, over expression of *yidC* was found to be inhibitory to *E. coli* growth (data not shown). Hence antisense based method is suitable for screening specific inhibitors against such proteins whose overexpression leads to cell death. The RNA silencing approach applied in this study offers a means to speedy identification of putative YidC inhibitors. Antimicrobial synergies between mRNA- and protein-level inhibitors have been described previously [39]. Synthetic RNA silencers have emerged as a novel class of antibacterials [42]. Similar antisense oligonucleotides directed against *yidC* mRNA can be designed and used in combination with essential oils eugenol and carvacrol for antibacterial therapy. Our studies taken together with recent research by other groups [16], [30] establish the usefulness of antisense RNA technology in *E. coli* to explore mode of action of antibacterial agents. Using this technology, we have shown for the first time that *yidC* is a therapeutic target of antibacterial essential oils eugenol and carvacrol in *E. coli*.
